# Assessment of the effect of local application of amifostine on acute radiation-induced oral mucositis in guinea pigs

**DOI:** 10.1093/jrr/rru024

**Published:** 2014-04-04

**Authors:** Chang Jiang Li, Sheng Zi Wang, Shu Yi Wang, Yan Ping Zhang

**Affiliations:** 1 Department of Otorhinolaryngology–Head and Neck Surgery, Fudan University Affiliated Eye, Ear, Nose and Throat Hospital, Fenyang Road 83, Xuhui District, Shanghai 200031, China; 2 Department of Radiation Oncology, Fudan University Affiliated Eye, Ear, Nose and Throat Hospital, Fenyang Road 83, Xuhui District, Shanghai 200031, China; 3 Department of Pathology, Fudan University Affiliated Eye, Ear, Nose and Throat Hospital, Fenyang Road 83, Xuhui District, Shanghai 200031, China; 4 Central Laboratory, Fudan University Affiliated Eye, Ear, Nose and Throat Hospital, Fenyang Road 83, Xuhui District, Shanghai 200031, China

**Keywords:** amifostine, local application, intercellular adhesion molecule-1 (ICAM-1), oral mucositis, guinea pig

## Abstract

The aim of present study was to assess the radioprotective effects of the local application of amifostine to treat acute buccal mucositis in guinea pigs. A total of 32 guinea pigs were randomized into four groups: (Group A) topically administered 50 mg of amifostine plus radiotherapy (RT); (Group B) 100 mg amifostine plus RT; (Group C) normal saline plus RT; and (Group D) normal saline plus sham RT. The opportunity for administration was 15 min before irradiation. When administered, the cotton pieces that had been soaked with 0.5 ml amifostine solution or saline were applied gently on the buccal mucosa of each guinea pig for 30 min. The animals in Groups A, B and C were irradiated individually with a single dose of 30 Gy to the bilateral buccal mucosa. Eight days after irradiation, the animals were scored macroscopically; they were then euthanized, and the buccal mucosal tissues were processed for hematoxylin–eosin staining and ICAM-1 immunohistochemical analysis. In Groups A and B, the mean macroscopic scores were 2.9 ± 0.6 and 2.4 ± 1.1, respectively. There was no significant difference between the two groups (*P* > 0.05). However, when they were separately compared with Group C (4.4 ± 0.7), a noticeable difference was obtained (*P* < 0.05). No mucositis was observed in Group D. Comparisons of the expression of ICAM-1 were in agreement with the macroscopic data. Histologically, superficial erosion, exudate and ulcer formation were all observed in the RT groups; only the severity and extent were different. The microscopic observations in the amifostine-treated groups were better than in Group C. The results demonstrated that topical administration of amifostine to the oral mucosa is effective treatment of acute radiation-induced mucositis.

## INTRODUCTION

Radiotherapy (RT) plays an important role in the management of head and neck cancer (HNC). The majority of new cases of invasive HNC are primarily treated with RT as an adjunct to surgery, in combination with chemotherapy, or as palliation [1]. With existing treatment, the tissue destruction and functional alterations in the oral cavity—especially radiation-induced oral mucositis (ROM)—are an inevitable problem when patients with HNC receive RT [2]. Trotti *et al.* reported that 11% of these patients had RT regimens that were interrupted or modified [3]. Additionally, acute severe ROM is associated with significant discomfort and impairment of the patient's ability to eat and swallow. ROM is also related to breaks in treatment, the placement of feeding tubes, and hospitalization [4]. Thus, it is of great importance and necessity to prevent and treat radiation-induced oral mucositis. However, although some measures have been encouraged (such as the use of an oral rinse, topical application of disinfecting agents and antimicrobials, and the use of anti-inflammatory and analgesic agents as well as some non-pharmaceutical products), there are no current clinical guidelines for ROM treatment.

The adhesion of inflammatory cells to endothelial cells is considered to be involved in the process of radiation-induced damage. Intercellular adhesion molecule-1 (ICAM-1) is thought to play an important role in this process. ICAM-1 is constitutively present on the cell surface of a wide variety of cell types, including fibroblasts, leukocytes, keratinocytes and endothelial cells, and its expression is increased after irradiation (IR) [5]. Hallahan *et al*. have demonstrated that ICAM-1 is required for inflammatory cell infiltration into radiation-induced pneumonitis and that ICAM-1 knockout mice have no increase in inflammatory cell infiltration into the lung in response to thoracic irradiation. Hallahan *et al*. also found a radiation dose-dependent increase in ICAM-1 expression in the endothelium [6]. To some extent, the level of ICAM-1 is associated with the severity of the radiation-induced inflammatory reaction, and its expression seems to be useful as a reference for the inflammatory response. In our study, we continue to explore the relationship between the expression of ICAM-1 and the radiation-induced inflammatory reaction.

Amifostine [S-2-(3-aminopropylamino) ethyl dihydrogen phosphorothioate], previously known as WR-2721, is a pharmacologically inactive prodrug that must be converted *in vivo* by alkaline phosphatase to an active sulfhydryl compound (WR-1065). WR-2721 not only effectively prevents and reduces injury from chemotherapy, it also protects from the effects of irradiation [7]. In 1999, the cytoprotective effects of amifostine led to its approval by the Food and Drug Administration (FDA) for the prevention of xerostomia in HNC patients receiving postoperative radiotherapy. This preventative role is highly selective in that it only protects normal tissues and not the tumor. There are many reports that the intrarectal application of amifostine can protect the rectum from radiation-induced injuries, but there are few studies on the local application of amifostine in the oral cavity. Therefore, if the topical administration of amifostine to the oral mucosa is effective, it will be helpful for patients suffering from ROM. The aim of the present study is to evaluate the radioprotective effects of the local administration of a dose of amifostine on irradiated mucosa.

## MATERIALS AND METHODS

### Study design

Our study protocol was reviewed and approved by the Laboratory Animal Ethical Board of the Eye, Ear, Nose and Throat Hospital of Fudan University. A total of 32 guinea pigs weighing ∼300 g each were used for the experiment. The animals were subjected to one week of preliminary conditioning. During that period and the follow-up period, they were housed under conditions with controlled humidity (30–50%) and temperature (21–24°C). A 12:12-h light–dark rhythm was maintained with the lights on between 06:00 and 18:00 h. The animals had access to food and water *ad libitum*.

The guinea pigs were assigned randomly to four groups (Groups A, B, C and D), with eight animals per group. The guinea pigs in Groups A and B were administered topical amifostine (50 mg and 100 mg, respectively) on the buccal mucosa before irradiation (RT + 50/100 mg Amifostine). The guinea pigs in Group C received physiologic saline at the same dose before irradiation (RT + saline). Group D was given physiologic saline before a sham irradiation (sham RT + saline).

### Drug dose and administration

WR-2721 was obtained from Merro Pharmaceutical Company Ltd (Dalian, China). The drug was dissolved in cold normal saline to final concentrations of 100 g/l and 200 g/l. Samples of the solution (0.5 ml) were removed using a medical syringe and placed on pieces of sterile cotton. Each piece of cotton contained 50 mg or 100 mg of amifostine.

Before the experiment, the animals fasted for 8 h. They were anesthetized using 50 mg/kg ketamine and 12.5 mg/kg xylazine administered via intramuscular injection. The duration of anesthesia was ∼2 h. A medical eyelid-opening device was used as a mouth gag against the teeth of the upper and lower jaw to hold the oral cavity open. The cotton pieces that had been soaked with 0.5 ml amifostine solutions were applied gently on the buccal mucosa of Groups A and B for 30 min; thus, the interaction time between the drug and buccal mucosa was 30 min. The guinea pigs in Groups C and D were given saline-containing cotton pieces. The guinea pigs were irradiated under a linear accelerator 15 min after removing the cotton pieces.

### Irradiation

**Fig. 1.**
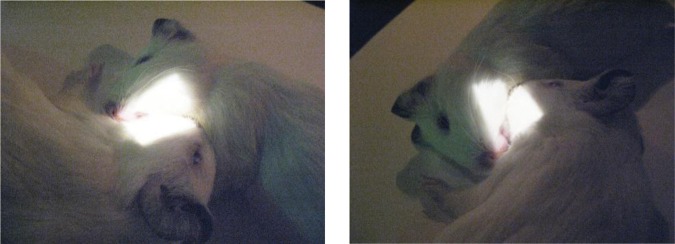
The guinea pig's positions at the time of irradiation.

The guinea pigs in Groups A, B and C were irradiated individually with a single dose of 30 Gy to the bilateral buccal mucosa using a Varian linear accelerator 6-MV X-ray (Varian 2300 CD, Palo Alto, California, USA). The source–skin distance was ∼80 cm. The dose rate was ∼2.5 Gy/min. During irradiation, the animals were under anesthesia. Two guinea pigs were simultaneously irradiated, one in the right lateral position and the other in the left position. The heads stayed close to each other. Correct positioning of the fields was controlled for each individual guinea pig using a therapy simulator. Ionizing radiation was specifically targeted at a 3 × 4 cm open buccal field (each was 3 × 2 cm). Although there was some difference between the bilateral buccal mucosa distance from the irradiation source, the number of animals in each group receiving irradiation was equally distributed, and there was no significant difference between our experimental results for each side. Hence, we merged the data together to analyze it. The animals were closely observed until recovery from anesthesia and returned to their cages. The control group received an equal-field sham irradiation and was treated with saline. In this model, no other systemic effects were elicited in the animals because of direct targeting of the radiation field (Fig. 1).

### Euthanasia

Guinea pigs from each group were weighed, scored and then euthanized on Day 8 after the irradiation or sham irradiation. The study endpoint was typical of the ulceration form phase in the development of ROM in the Parkins *et al*. and Sonis *et al*. model system [8, 9]. Euthanasia was performed by cardiac exsanguination via ketamine and xylazine anesthesia. The buccal specimens were excised for histopathologic evaluation.

### Scoring of mucosal reactions

The guinea pigs were observed every day, and mucosal reactions were scored using a scoring system described by Sonis *et al*. [9]. On Day 8 post-irradiation, film was developed using a digital camera, and the resulting photos were randomly numbered and then scored in a blinded fashion by two observers. A 0–5 scoring system was used (Table 1). The reported scores represent the average of the observations from the two, blinded observers.

**Table 1.** 0–5 scoring system

Score	Damage degree of oral mucosa induced by irradiation
0	Normal mucosa
1	Erythema and vasodilation
2	Severe erythema and vasodilation, with erosion of superficial aspects of mucosa leaving denuded areas with decreased stippling of the mucosa
3	Severe erythema, vasodilation, and the formation of ulcers in one or more places (in which the cumulative size of ulcers involved was ∼25% of the buccal mucosa), and pseudomembrane formation was evident
4	Severe erythema and vasodilation, and the formation of ulcers in one or more places (in which the cumulative size of the ulcers involved was ∼50% of the buccal mucosa), together with a loss of mucosal pliability
5	Diffuse, extensive ulceration.

### Histopathologic analysis

All buccal specimens were fixed in 4% paraformaldehyde, and representative portions were embedded in paraffin after the routine processing of the tissues. Four-micrometer sections were obtained from each paraffin block and stained with hematoxylin and eosin. Slides were examined twice (by two different pathologists) under a light microscope (Nikon Eclipse E600) in a blinded manner. For ICAM-1 immunostaining, two sections (4 mm) of each buccal mucosa were mounted onto a slide. The sections were cleared in xylene and hydrated through a descending alcohol series to distilled water. Internal peroxidase activities were suppressed with 1% H_2_O_2_ in methanol for 10 min. An antigen retrieval step was performed using EDTA, pH 8.0, 20 min at 100°C. Sections were preincubated with normal bovine serum (Boster ABC Kit, Wuhan, China) to prevent nonspecific binding and were then incubated overnight at 4°C with anti-mouse ICAM-1 antibody at a 1:100 dilution (Monoclonal Anti-mouse ICAM-1, Abcam, UK). The primary antibody was only added to one of the two sections on each slide, whereas the other was incubated with PBS and served as a control. After washing in PBS, the slides were incubated with the secondary antibody for 15 min at 37°C, followed by incubation with the ABC complex. DAB was used for color development. The intensity of the ICAM-1 signal in the cells in one microscopic field was assessed semi-quantitatively. An arbitrary score ranging from 0–3 was applied, with 0 representing a lack of signal, 1 a weak staining, 2 a mild staining and 3 a maximum signal. The intensity of the ICAM-1 signal was determined at a magnification of ×20.

### Statistical analysis

Results are expressed as the means or numbers. The differences in the macroscopic scores between the four groups were analyzed using the SPSS17.0 statistical package. In the immunohistochemical scores, the means and the standard deviations of the mean for the staining signal were computed for each group based on the individual means for each animal (using the SPSS17.0 statistical package). The Student-Newman-Keuls test was used to compare any two of several samples. A *P*-value < 0.05 was considered statistically significant.

## RESULTS

**Fig. 2.**
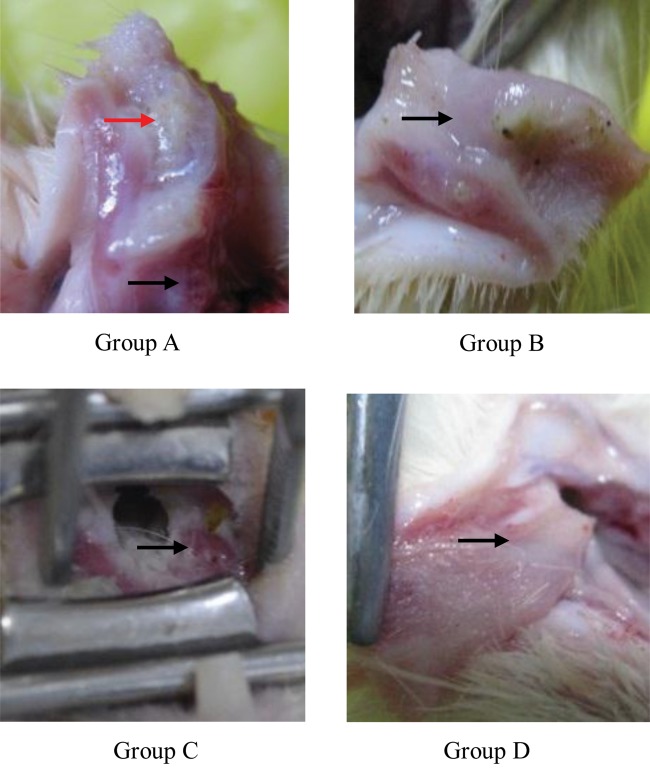
Representative and typical macroscopic damage of the buccal mucosa of guinea pigs on Day 8 after irradiation. **Group A**. Moderate to severe mucosal erosions (black arrow), and excoriations and superficial small ulcers (red arrow) were observed. **Group B**. Mild to moderate mucosal erosions, excoriations and occasional, irregular, superficial small ulcers were observed. **Group C**. Severe excoriations and vasodilation, bleeding, and extensive, large and deep ulcerations were observed. **Group D**. Normal mucosa.

Guinea pigs were selectively irradiated with a single dose of 30 Gy on the buccal mucosa of two sides. No mortality was observed in any of the groups. Three days after irradiation, the guinea pigs of three experimental groups (Groups A, B and C) had reduced food intake. On Day 8, they lost weight and had wetness surrounding the mouth or oral cavity. Monitoring of the ROM in the guinea pigs (*n* = 8) was performed using the macroscopic 0–5 scoring system. On Day 8 post-irradiation, the mean mucositis score in Group A was 2.9 ± 0.6, while local treatment with amifostine at 100 mg (Group B) reduced the mean mucositis score to 2.4 ± 1.1, which was not significant (*P* > 0.05). However, topical administration of normal saline (Group C) resulted in the most severe mucositis—a score of 4.4 ± 0.7, which was significantly higher than Groups A and B (*P* < 0.05). In Group D, no mucositis was observed (Table 2, Fig. 2).

**Table 2.** The macroscopic scores of the four groups

Groups	Scores (the mean ± sd)	Buccal mucosal samples (*n*)	Pairwise comparison
**A (50 mg amifostine + RT)**	2.9 ± 0.6	16	*P* > 0.05 vs B
**B (100 mg amifostine + RT)**	2.4 ± 0.1	16	*P* < 0.05 vs C
**C (Normal saline + RT)**	4.4 ± 0.7	16	*P* < 0.05 vs A
**D (Normal saline + sham RT)**	0.0 ± 0.0	16	*P* < 0.05 vs A, B, C

**Fig. 3.**
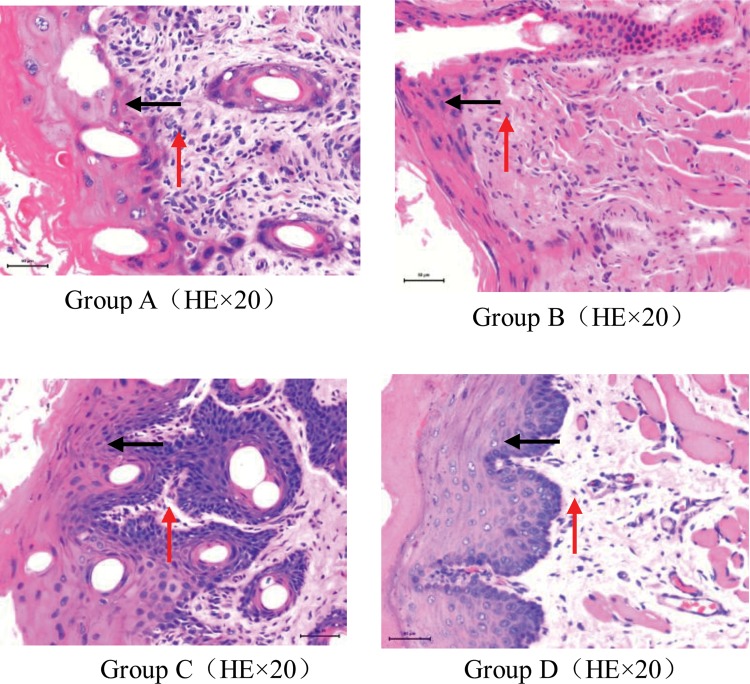
Representative and typical microscopic changes in the buccal mucosa of guinea pigs in each group on Day 8 after irradiation. **Group A.** The epithelial layer was destroyed, and the structure was not clear (black arrow). Epithelium hyperplasia (black arrow), interstitial proliferation, and variable inflammatory cell infiltration were observed (red arrow). Sometimes, ulcer formation was observed. **Group B.** Destruction of the epithelium and moderate inflammatory cell infiltration in the stroma were observed, however, the degree of injury of the tissues was less than in Group A. **Group C.** The epithelial structure was heavily damaged. Ulcer formation, significant dysplasia of the epithelial and interstitial tissues, and substantial inflammatory cell infiltration were observed. **Group D.** Intact, healthy mucosa.

Transverse sections (4 mm in thickness) of the paraffin-embedded buccal mucosa specimens of guinea pigs were stained with hematoxylin and eosin (H&E) and examined with a light microscope (×20) on Day 8. The gross morphology of the layers of buccal mucosa epithelium and submucosa was similar in the groups that received different amifostine doses (50 mg and 100 mg). In Group A (RT + 50 mg amifostine), the following moderate histological alterations were observed: cell infiltration with lymphocyte prevalence, edema, erythema, bleeding, vasodilation, superficial erosion, exudate and superficial small discrete ulceration in a minority of guinea pigs. However, there was an absence of abscess. There was medium breakdown in the integrity of the mucosal epithelium. Hyperplasia of the epithelium and stroma were found in pathology. Group B (RT + 100 mg amifostine) had fewer effects than Group A. In these animals, the histology was consistent with oral ulcerations involving inflammatory reactions with various immune system cells. The difference in the histological lesions between Group A and Group B was not remarkable. In Group C (RT + saline), corresponding to severe mucositis on Day 8 post-irradiation, there was serious edema, accentuated vascular dilatation and intense cellular infiltration with lymphocyte prevalence by light microscopy. Furthermore, large, extensive and deep ulcers could be observed in most of the animals. Additionally, there was severe epithelium dysplasia and stroma proliferation. In contrast with the saline-treated animals receiving RT, amifostine (topically administered at 50 mg and 100 mg) significantly reduced the histological alterations observed in the experimental oral mucositis. In Group D, no histological lesions were observed (Fig. 3).

**Fig. 4.**
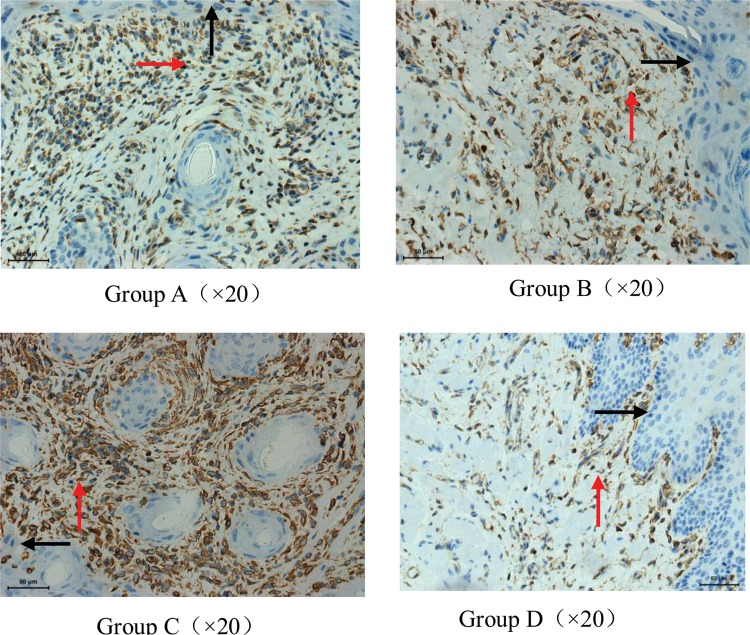
The intensity of the expression of ICAM-1 of the buccal mucosa and stroma of guinea pigs on Day 8 after irradiation. Weak constitutive ICAM-1 expression (staining intensity 1) in the epithelium (black arrow) and stroma (red arrow) of a sham-irradiated control buccal mucosa was shown in **Group D**. **Group C**. Increased ICAM-1 expression (staining intensity 3) in the epithelium and stroma on Day 8 after irradiation. The increase in ICAM-1 expression was accompanied by substantial leukocyte infiltration, epithelium hyperplasia and stroma proliferation. The staining intensity of ICAM-1 expression in the vast majority of specimens in **Group A and B** was at a level of 2, and a few were stained as 3 or 1.

In the buccal mucosa of the sham RT plus saline group (Group D), epithelial and stroma cells, mainly lymphocytes, constitutively expressed ICAM-1 at low levels, and the group displayed scores of 0.63 ± 0.52. In the RT plus saline group (Group C), irradiation resulted in a clear increase in the epithelial ICAM-1 expression, which was also accompanied by leukocyte infiltration on Day 8 post-irradiation, and the group scored an average of 2.88 ± 0.35. The group receiving RT plus topical administration of 50 mg amifostine (Group A) had similar ICAM-1 levels (2.13 ± 0.84) as the group receiving RT plus topical administration of 100 mg amifostine (Group B) (2.00 ± 0.76). However, there was a significant difference between Groups A and C (*P* < 0.05). Additionally, the expression of ICAM-1 in Group B was markedly lower than in Groups C and D (*P* < 0.05). The expression of ICAM-1 in Group C was higher than in Group D (*P* < 0.05) (Table 3, Fig. 4).

**Table 3.** The scores of the expression of ICAM-1 in the four groups

Groups	**Scores (the mean ± standard deviation)**	**Buccal mucosal samples (*****n*****)**	**Pairwise comparison**
**A (50 mg amifostine + RT)**	2.13 ± 0.84	16	*P* > 0.05 vs B
**B (100 mg amifostine + RT)**	2.00 ± 0.76	16	*P* < 0.05 vs C
**C (Normal saline + RT)**	2.88 ± 0.35	16	*P* < 0.05 vs A
**D (Normal saline + sham RT)**	0.63 ± 0.52	16	*P* < 0.05 vs A, B, C

## DISCUSSION

Radiotherapy of advanced cancers in the head-and neck region is frequently associated with oral mucositis, which often necessitates interruption of the treatment to allow for healing of the mucosal reaction [10]. Thus, it is critical for oncologists to prevent and treat this harmful complication. Amifostine has long been known to be useful as a radiation protectant in patients receiving RT. Amifostine is a prodrug that is dephosphorylated *in vivo* by alkaline phosphatase to become the active cytoprotective thiol metabolite, WR-1065, the form of the drug that is taken up into cells and that is the major cytoprotective metabolite. The selective protection of certain tissues of the body is believed to be due to the higher alkaline phosphatase activity, higher pH and vascular permeation of normal tissues [11]. Amifostine, administered intravenously, reduces the incidence of acute and late xerostomia in HNC patients who receive radiotherapy. However, there are many systemic side-effects associated with intravenous administration. Several studies have been performed to investigate the use of other drug delivery methods, such as subcutaneous (SC) and rectal routes. Recently, some authors reported that intrarectal administration of amifostine may reduce radiation damage and have superior cytoprotective efficacy in acute radiation rectal mucositis compared with subcutaneous administration [12, 13, 14].

Before this study, we performed a feasibility study on the pharmacology of local application of amifostine (WR-2721). We topically administered 50 mg or 100 mg of WR-2721 to the buccal mucosa in guinea pigs and detected the drug concentration in the tissues and plasma, using HPLC-MS/MS. The local application of WR-2721 in the oral cavity is feasible and can lead to relatively large quantities of amifostine and WR-1065 in the buccal mucosal tissues within 15 min of administration (the time of drug administration was 30 min), while systemic absorption is negligible. Therefore, in this study, we also chose this time-point for irradiation [15]. We determined whether the concentrations of amifostine or WR-1065 in tissues can be efficacious on ROM. We used the experimental model of ROM established by Parkins and Sonis *et al*. and reduced the irradiation dose to 30 Gy; this still produced ulcerative mucositis, such as that described by Parkins and Sonis *et al*. An earlier published study showed that 50 mg of amifostine applied topically to the buccal mucosa of mice can reduce the severity of acute mucositis [16], but there are some differences between their study and ours. We used a different irradiation regimen. We adopted a single fraction of 30 Gy, while they administered a total of 24 Gy in 4 fractions, with an 8-h interval between each fraction. However, the experimental result is in agreement with ours.

We also observed histopathologic differences between the groups of guinea pigs. In irradiated plus saline-treated animals on Day 8, the epithelial layer was destroyed, with significant proliferation, accompanied by ulceration and an increase in inflammatory cells. In contrast, although there was some distortion and hyperplasia of the epithelial cell layer and stroma in amifostine-treated animals, they remained relatively intact and the pathological changes were less apparent. These microscopic aspects of the inflammation were in agreement with the macro-observations.

ICAM-1 is thought to play an important role in the radiation-induced inflammation reaction. An increase in ICAM-1 expression in response to irradiation has been described in several tissues in humans as well as in experimental animals. Ikeda *et al*. found that ICAM-1 upregulation in endothelial cells played an important role in the development of radiation-induced colonic ulcers [17]. Gaugler *et al*. reported that radiation-induced increase in ICAM-1 expression on HUVEC correlated with augmented adhesion of neutrophils on irradiated endothelial cells [18]. And Gaber *et al.*'s research results showed that the molecular response of the brain to single-dose irradiation was rapid, while its response to fractionated irradiation was slow [19]. Additionally, another research group demonstrated that endothelial ICAM-1 was involved in the pathogenesis of radiation-induced urinary bladder effects [20]. In the aforementioned studies, ICAM-1 participated in the irradiation-induced inflammation in various tissues and organs. Many of these findings are consistent with those of our experiments, in which 8 d after a single irradiation of 30 Gy to the buccal mucosa of guinea pigs, the expression of ICAM-1 proteins increased remarkably in the mucosal epithelium and leukocytes in the stroma in the RT groups. Our study also indicates that ICAM-1 is associated with the severity of radiation-induced inflammation. In the saline-treated guinea pigs receiving RT, ICAM-1 expression in the mucosal epithelial layer and leukocytes in the stroma was significantly higher than for amifostine-treated animals. At the same time, the ROM damage was distinctly worse in Group C than in Groups A and B. These data suggest that ICAM-1 may mediate the interaction in this process.

## CONCLUSION

In conclusion, this is the first animal study that demonstrates that a local application of amifostine to the oral mucosa has a therapeutic effect on acute ROM. This study used a single fraction scheme and demonstrated that the expression of ICAM-1 protein is associated with the radiation damage. The local application of amifostine can reduce but not completely ameliorate the degree of ROM and can weaken the expression of ICAM-1 at the macroscopic and histological levels. ICAM-1 participates in the acute radiation-induced inflammatory reaction and may be an index for the radiation-induced inflammatory reaction.

## FUNDING

Funding to pay the Open Access publication charges for this article was provided by Health Science and Technology project of the Social Development Bureau of Pudong New Area (PW2009D-7) and Science and Technology Commission of Shanghai Medical bootstrap class project (09411962000).
